# Supplementary figures and images for: Interrogation of Essentiality in the Reconstructed Haemophilus influenzae Metabolic Network Identifies Lipid Metabolism Antimicrobial Targets: Preclinical Evaluation of a FabH β-Ketoacyl-ACP Synthase Inhibitor
Source: mSystems. 2022 Mar 16;7(2):e01459-21. doi: 10.1128/msystems.01459-21 (PMC9040583; doi:10.1128/msystems.01459-21)

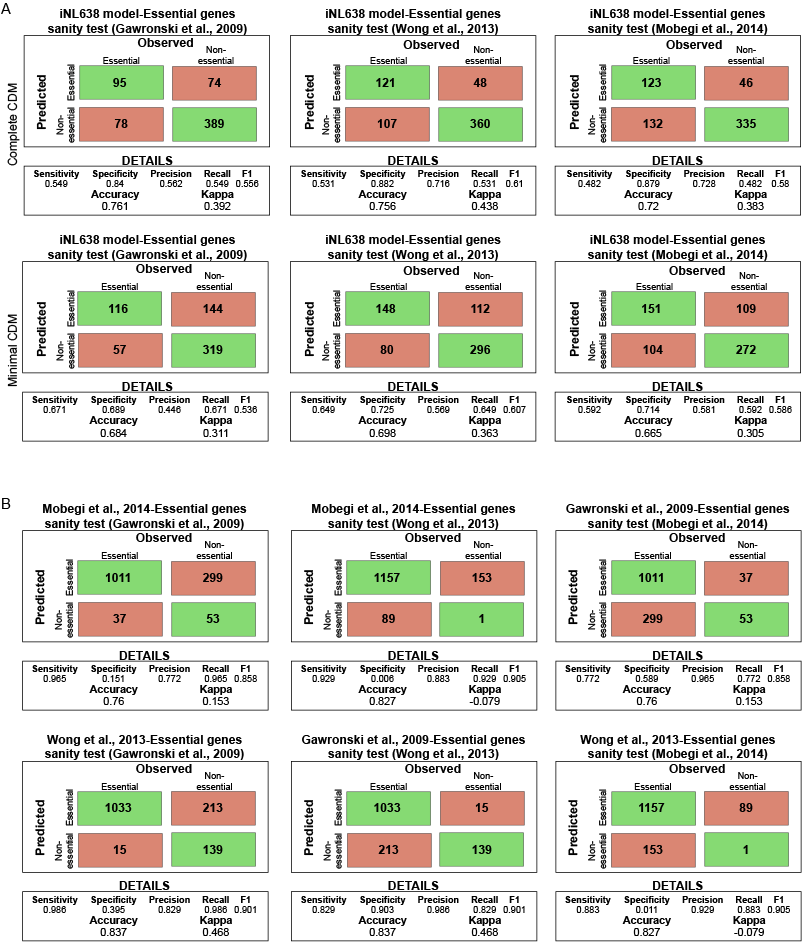

Supplement: FIG S1 [file msystems.01459-21-sf001.tif]

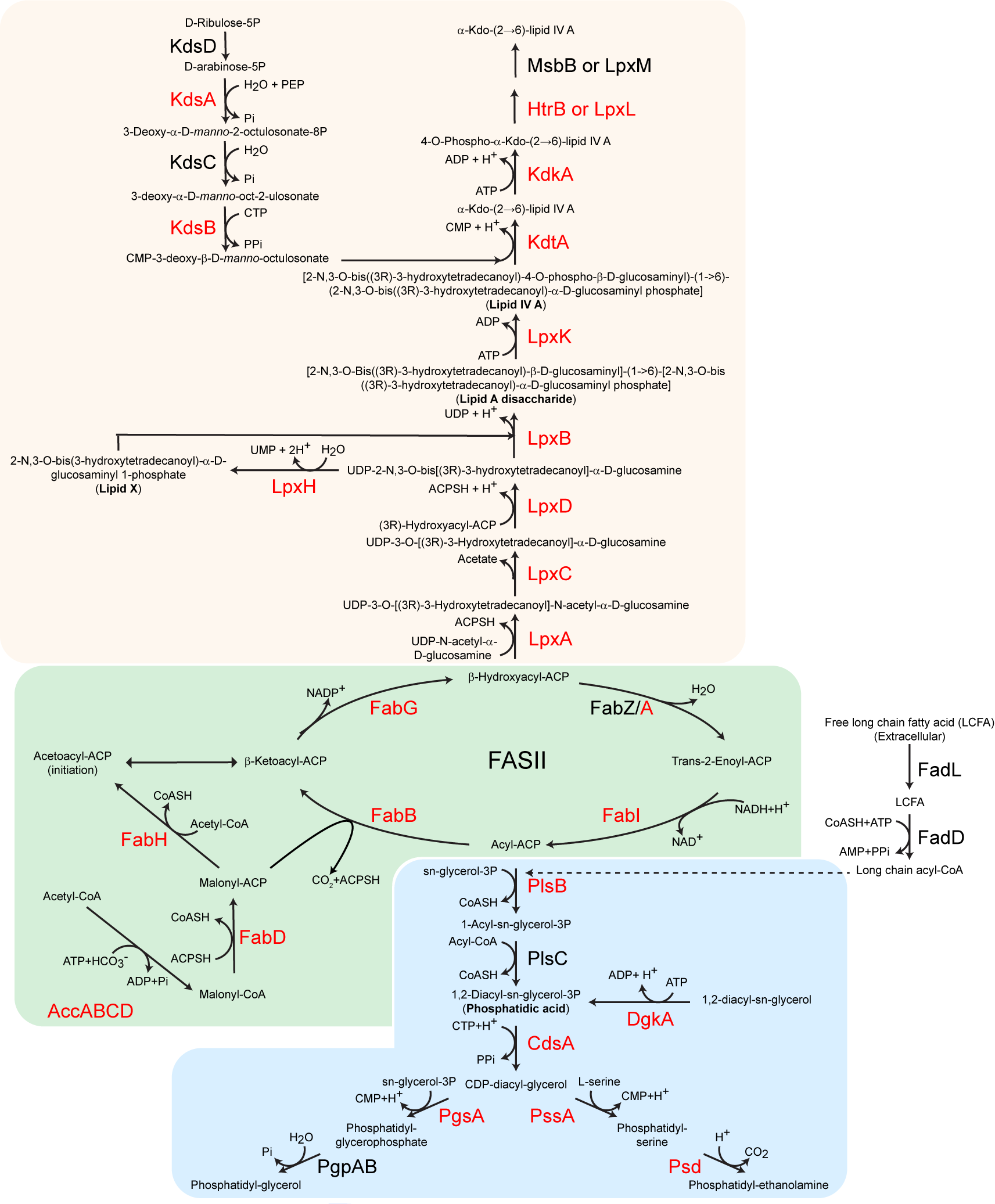

Supplement: FIG S2 [file msystems.01459-21-sf002.tif]

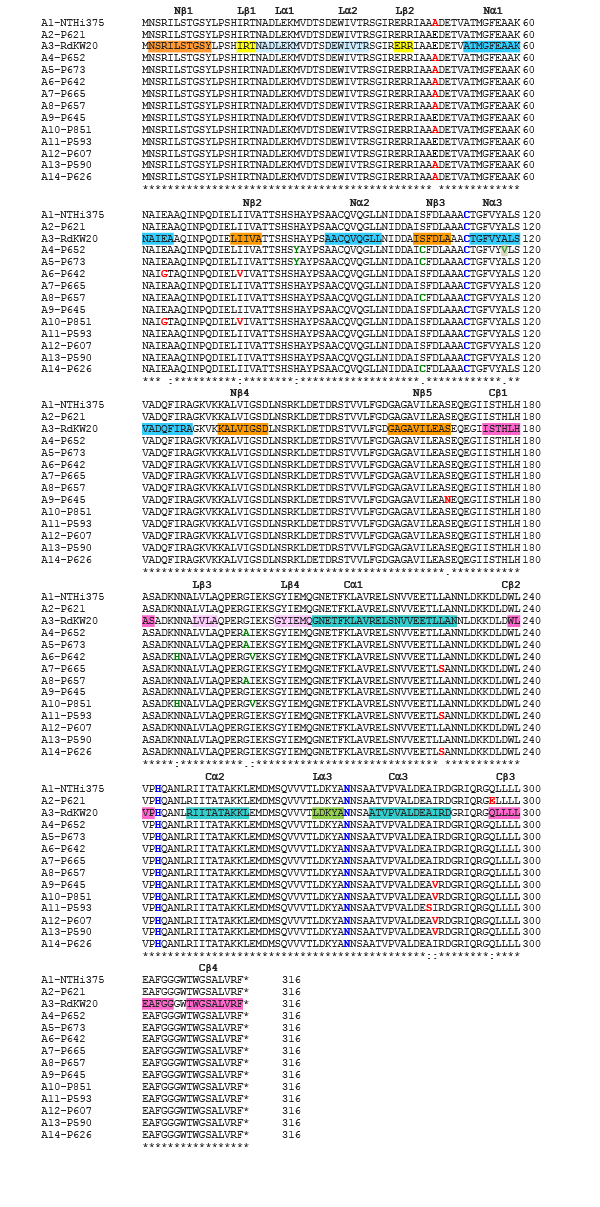

Supplement: FIG S3 [file msystems.01459-21-sf003.tif]

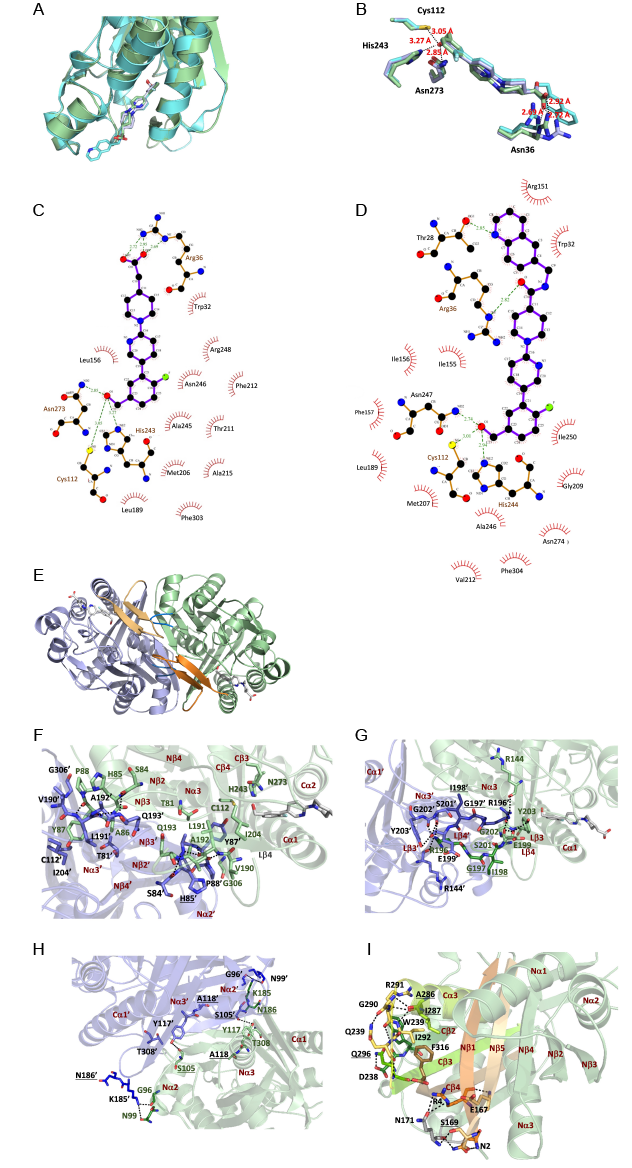

Supplement: FIG S4 [file msystems.01459-21-sf004.tif]

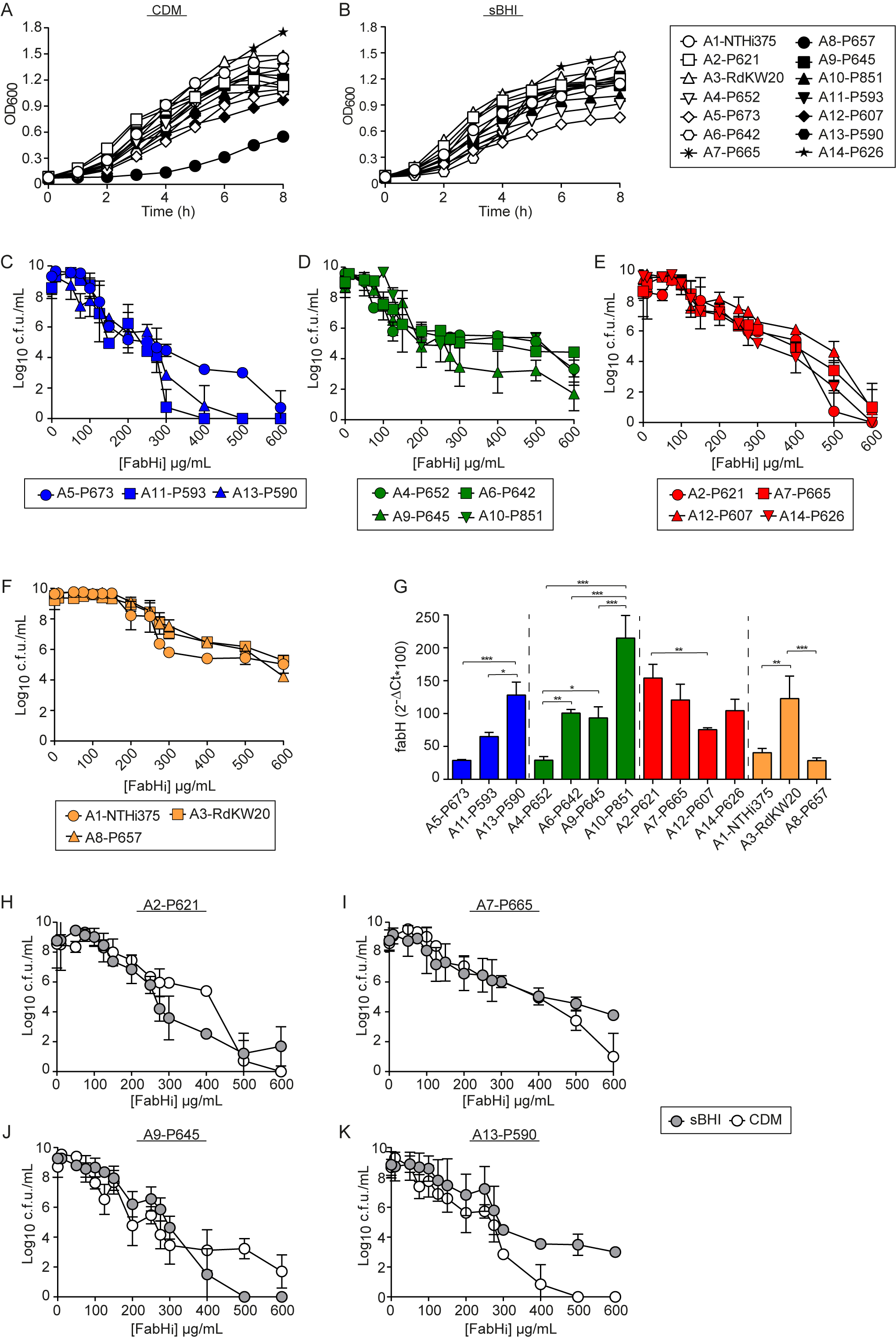

Supplement: FIG S5 [file msystems.01459-21-sf005.tif]

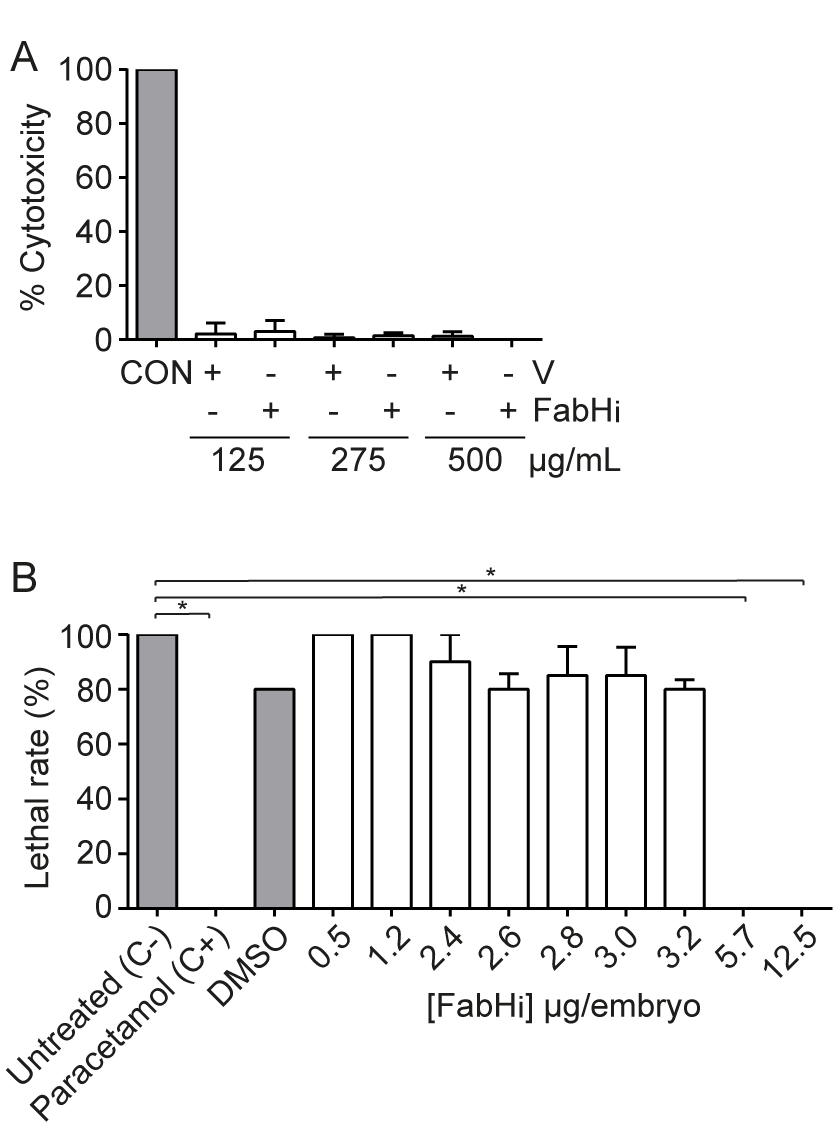

Supplement: FIG S6 [file msystems.01459-21-sf006.tif]
