# Supplementary material for: Interrogation of Essentiality in the Reconstructed Haemophilus influenzae Metabolic Network Identifies Lipid Metabolism Antimicrobial Targets: Preclinical Evaluation of a FabH β-Ketoacyl-ACP Synthase Inhibitor
Source: mSystems. 2022 Mar 16;7(2):e01459-21. doi: 10.1128/msystems.01459-21 (PMC9040583; doi:10.1128/msystems.01459-21)
Supplement: TABLE S1 [file msystems.01459-21-st001.docx]

**Table S1.** Bacterial cultures dry weight.

| **OD_600_** | **Collected volume (mL)** | **Dry pellet weight (mg)** | **mg/mL** |
| --- | --- | --- | --- |
| 0.3 | 45 | 12.05 | 0.27 |
| 0.4 | 30 | 11.9 | 0.40 |
| 0.8 | 15 | 10.7 | 0.71 |
