# Supplementary material for: Interrogation of Essentiality in the Reconstructed Haemophilus influenzae Metabolic Network Identifies Lipid Metabolism Antimicrobial Targets: Preclinical Evaluation of a FabH β-Ketoacyl-ACP Synthase Inhibitor
Source: mSystems. 2022 Mar 16;7(2):e01459-21. doi: 10.1128/msystems.01459-21 (PMC9040583; doi:10.1128/msystems.01459-21)
Supplement: TABLE S2 [file msystems.01459-21-st002.docx]

**Table S2.** Primers used in this study.

| **Primer**  **name** | **Primer**  **ID** | **Sequence (5’-3’)** | **Purpose** | **Reference** |
| --- | --- | --- | --- | --- |
| *fabH*-F1 | 1702 | AAATCAGCTTGAGCGTTTGGCGGAATC | Gene inactivation | This study |
| *fabH*-R1 | 1703 | AAAGAAGGGAACCCAATTTGTTCT | Gene inactivation | This study |
| *fabH*-F2 | 1704 | GATAGTCCATAGATTTTTTCCTTTTTAATCGCTTATCTTATTGAGTATGATTCCGGGGATCCGTCGACC | Gene inactivation | This study |
| *fabH*-R2 | 1705 | GCATCTCGATATAGCCAGATTTTTCTATACCACGTTCTGGCTGAGCTAAATGTAGGCTGGAGCTGCTTCG | Gene inactivation | This study |
| *fabH*-F | 1756 | TTATTCGTGCAGGCAAAGTG | qRT-PCR | This study |
| *fabH*-R | 1757 | AAGCGTGTAAATGGGTGGAG | qRT-PCR | This study |
| *fabH*-P851-P642-F | 1906 | TTATTCGTGCAGGCAAGGTG | qRT-PCR | This study |
| *fabH*-P851-P642-R | 1907 | AAGCGTGTAAATGGGTAGAA | qRT-PCR | This study |
| *gyrA*-qPCR-F2 | 1078 | ATATGTTGGTTGATGGGCAAGG | qRT-PCR | (20) |
| *gyrA-*qPCR-R2 | 1079 | GGCGAGAAATTGACGGTTTCT | qRT-PCR | (20) |
